# Development of alternative versions of the Logical Memory subtest of the WMS-R for use in Brazil

**DOI:** 10.1590/1980-57642015DN92000008

**Published:** 2015

**Authors:** Silvia Adriana Prado Bolognani, Monica Carolina Miranda, Marjorie Martins, Patricia Rzezak, Orlando Francisco Amodeo Bueno, Candida Helena Pires de Camargo, Sabine Pompeia

**Affiliations:** 1 Centro Paulista de Neuropsicologia, Associação Fundo de Incentivo à Pesquisa.; 2 Departamento de Psicobiologia, Universidade Federal de São Paulo.; 3 Lim-21, Instituto de Psiquiatria, Universidade de São Paulo.; 4 Instituto de Psiquiatria, Universidade de São Paulo.

**Keywords:** logical memory, episodic memory, prose recall, memory test, stories

## Abstract

The logical memory test of the Wechsler Memory Scale is one of the most frequently used standardized tests for assessing verbal memory and consists of two separate short stories each containing 25 idea units. Problems with practice effects arise with re-testing a patient, as these stories may be remembered from previous assessments. Therefore, alternative versions of the test stimuli should be developed to minimize learning effects when repeated testing is required for longitudinal evaluations of patients.

**Objective:**

To present three alternative stories for each of the original stories frequently used in Brazil (Ana Soares and Roberto Mota) and to show their similarity in terms of content, structure and linguistic characteristics.

**Methods:**

The alternative stories were developed according to the following criteria: overall structure or thematic content (presentation of the character, conflict, aggravation or complements and resolution); specific structure (sex of the character, location and occupation, details of what happened); formal structure (number of words, characters, verbs and nouns); and readability.

**Results:**

The alternative stories and scoring criteria are presented in comparison to the original WMS stories (Brazilian version).

**Conclusion:**

The alternative stories presented here correspond well thematically and structurally to the Brazilian versions of the original stories.

## Abstract (pt)

O teste Memória Lógica da Escala Wechsler de Memória é uma das tarefas mais empregadas de avaliação de memória e é composto de duas histórias sobre diferentes personagens que contém 25 itens. A repetição destas histórias para a mesma pessoa em diferentes ocasiões leva a uma melhora nos escores por efeito de familiaridade com seu conteúdo. Portanto, versões alternativas deste teste devem ser construídas de modo a permitir que o teste seja repetido em situações como a avaliação longitudinal de pacientes.

**Objetivo:**

Apresentar 3 histórias alternativas a cada uma das versões das histórias originais mais empregadas no Brasil (Ana Soares e Roberto Mota).

**Métodos:**

As histórias alternativas foram construídas obedecendo aos seguintes critérios: estrutura geral ou conteúdo temático (apresentação do personagem; conflito; agravante ou complemento; resolução); estrutura específica (sexo do personagem; localidade e ocupação, detalhes da ocorrência); estrutura formal (número de palavras, caracteres, verbos e substantivos); e legibilidade.

**Resultados:**

São apresentadas as histórias alternativas e critérios de correção em comparação às versões das histórias originais.

**Conclusão:**

as histórias alternativas apresentadas tem correspondência temática e estrutural com as versões brasileiras do teste original.

## INTRODUCTION

The Logical Memory task from the Wechsler Memory Scale (WMS)^1^ is one of the most frequently used tests for assessing episodic memory.^2^ This test is composed of two stories which include 25 units or ideas each, and are verbally presented by the examiner: one about a woman (presented first), and the other about a man.

Immediately after each story is presented (immediate recall), the test subject is asked to repeat what they remember as accurately as possible, without being given any clues (free recall).^1^ Thirty minutes later, subjects are asked to recall the story again (delayed recall). For both immediate and delayed recall, all correct items are summed giving a maximum score of 25 for each story. The WMS is currently in its 4^th^ edition,^3^ and since its previous version,^4^ the logical memory stories have gained an additional form of scoring. The 25-item scoring system was maintained (which counts specific units of the story), and a second scoring criteria was established, considering the recall of general topics (gist).^5^ For example, the original scoring system credits points for the recall of the character's exact name and surname (one point each); the additional scoring system now also grants a point if the subject recalls that the story was about a woman, even if her name is not remembered.

Because of its great value in memory assessment, the logical memory test is often used when re-testing is needed in clinical practice and longitudinal research. Changes in memory performance are often assessed after interventions such as surgeries,^6^ evolution of psychiatric disorders,^7^ as well as several types of therapeutic and pharmacological interventions.^8,9^ These studies involve the assessment of the same subjects on two or more separate occasions.

The logical memory test is known to be susceptible to practice effects or training, i.e., the repeated use of the same story increases the possibility that it is remembered on subsequent assessments (stimulus learning).^2,10^ Thus, there is an urgent need for alternative stories. However, few studies have developed alternative stimuli. This is the first step in proposing alternative versions of cognitive tasks, the second being the study of psychometric similarity between the versions in healthy participants and clinical populations. Various studies have suggested means of constructing alternative stories to those in the WMS as discussed below. In this study, the criteria from these scientific publications were used to construct the alternative versions of the task in Portuguese.

Morris, Kunka and Rossini^11^ presented an alternate version for each of the WMS-R stories that had equivalent characteristics in terms of their items (units of ideas), sentences, words per sentence, syllables per word, readability and subjective attributes (such as affective tone). In many clinical and experimental settings, however, more than one alternative story for each original may be required. Sullivan^2^ proposed six stories following similar criteria.^11^ The author then analyzed the comparability of stories in terms of number of words, characters, sentences and readability, as well as the performance of normal subjects in terms of immediate recall of these stories after oral presentation. Though these stories are generally similar, there were some differences between them, probably because the original stories of the WMS-R test are not equivalent in the parameters studied,^2^ and also differ in terms of ease of memorization.^12^

Two other publications have proposed alternative stories, applying similar methods to those adopted by the studies mentioned above.^2,11^ Schnabel^10^ published two alternative versions of the WMS-IV stories that were similar in structure, narrative, and readability. Moreover, he tested the compatibility of the WMS stories and the newly developed stories empirically in three large populations, comprising patients with depression, head injury, and a control group of healthy individuals. He demonstrated that performance regarding the original and the newly developed stories was interchangeable for all groups, both for immediate and delayed recall. A further study shows three short stories similar to each other, but these were not equivalent to the stories of the WMS.^13^

The use of the logical memory test in Brazil poses several major difficulties. To date, the test has not been published in Portuguese for any of the WMS versions. A free translation / adaptation of the pair of stories of the WMS-R (Ana Soares and Roberto Mota) was done for clinical and research purposes at one of the major neuropsychology centers in Brazil by Camargo et al.,^14^ but the stories in full and the scoring criteria were not published. One article carries one of the two stories in Portuguese (Ana Soares), but no scoring criteria were provided.^15^ These versions have been shared privately among memory specialists for years and are probably the most common prose recall tests used by Brazilian professionals in research and clinical work. Hence, there is no publication that includes both these adapted stories and their scoring criteria, nor scientific work on standardization or systematic adaptation of this instrument for use in Brazil.

There are stories for memory testing created in Portuguese from Brazil, but these are not equivalent to the any of the versions of the WMS, since they are shorter and have a very different thematic structure.^16,17^ Given that many Brazilian professionals have been using the logical memory test and may need to re-test patients, it would be useful if the alternative stories in Portuguese were constructed so as to be equivalent to those adapted by Camargo et al.^14^ from the stories of the WMS-R.

The aim of this study was to develop a set of alternative logical memory stories to those frequently used in Brazil (Ana Soares and Roberto Mota) in terms of their content, structure and linguistic characteristics. The development of the material was based on parameters proposed by previous authors,^2,10,11^ and also took into account the thematic structure of the original stories. The material presented here, however, does not show the psychometric equivalence of the stories, which was tested later.^18^

## METHODS

Three alternative stories for each of the stories in Portuguese (Ana Soares and Roberto Mota)^14^ were proposed by the authors following the criteria below, based on previous studies.^2,10,11^

1. **General structure of the plot:** each story involved the presentation of character, a conflict, an aggravation / complements to the main plot, and a resolution. The alternative stories to that of Ana Soares involved female characters whereas those of the Roberto Mota stories had male characters.
2. **Specific elements:** all stories presented the characters with a first name, surname, occupation and place of origin; the stories that were alternatives for the female story had numerals and the male ones had closure statements with a specific word that can be considered slightly out of the context that can be scored even if recalled alone (equivalent to the word 'shark' in the Roberto Mota story).
3. **Grammatical structure and details:** all stories were equivalent regarding the grammatical classes of words, number of words, letters, verbs and nouns per idea item and in total;
4. **Units of general topics (gist):** units that summarize the essential ideas of the excerpts of the story were considered, allowing little specific recollection to be granted a point on the secondary scoring system as mentioned in the introduction section. The units of general themes of the female stories were guided directly by those proposed by the WMS-III, which maintains the story equivalent to that of Ana Soares, with a total of 7 themes. The male stories had these units freely defined by the present authors, since the story that led to the Roberto Mota version was not maintained in the most recent versions of the test in its original English version; this secondary scoring has a total of 8 units for the stories of the male characters.
5. **Readability:** reading complexity was calculated with the Flesch Reading Ease Scores adapted for local use in Portuguese. Scores between 75 and 100 denote very easy to read, from 50 to 75 easy, 25 to 50 difficult and below 25 very difficult to read.^19^

Following the creation of the alternative stories, they were presented to a group of experts from the Psychology Department of the Universidade Federal de Sao Paulo, including memory researchers from different areas of Brazil (various sessions including more than 15 experts in each). This group of experts proposed changes that were necessary to make the stories easy to understand and equivalent to each other. The authors then determined the scoring criteria, which were subsequently evaluated by the group of memory experts. The final selection of acceptable responses was defined by consensus among the authors. The Appendix shows the scoring criteria for each story.

## RESULTS

The original stories in Portuguese^14^, and their organization according to thematic units, can be seen in Table 1. The new alternative stories are presented in Table 2, which also shows the equivalence to the original stories according to various parameters such as: thematic content, structure (sex of the character, their origin and profession, what happened and the resolution of the problem), grammatical details, as well as readability of stories, all of which were scored as easy to read according to the Flesch Readability Ease Scores (between 50 and 75).^19^ The Appendix shows the scoring criteria with the acceptable answers, both for the main score, which requires greater accuracy in recall, as well as for the secondary scoring, which evaluates the ability to remember general themes (gist). Examples of incorrect responses are also presented.

**Table 1:** Stories translated and adapted by Camargo et al.11 for use in Brazil and thematic structure (plot structure).

Female character (Ana Soares)			Male character (Roberto Mota)	
Structure	Story items		Structure	Story items
Character introduction	Ana		Character introduction	Roberto
	Soares			Mota
	do Sul			estava dirigindo
	do Paraná			um caminhão
	empregada			Mercedes
	como faxineira			numa rodovia
	em um prédio			à noite
	de escritórios			no Vale
Problem	relatou			do Paraíba
	na delegacia			levando ovos
	de polícia			para Taubaté
	que tinha sido assaltada		Problem	quando o eixo
	na Rua do Estado			quebrou.
	na noite anterior			O caminhão derrapou
	e roubada			caindo numa valeta
	em 150 Reais.			fora da estrada.
Aggravation	Ela disse que tinha 4		Aggravation	Ele foi jogado
	filhinhos			contra o painel
	o aluguel não tinha sido pago			e se assustou muito.
	e eles não comiam			Não havia trânsito
	há dois dias.			e ele duvidou que pudesse ser socorrido.
Resolution	Os policiais		Resolution	Naquele instante, seu radio amador
	tocados pela historia da mulher			tocou.
	fizeram uma coleta			Ele respondeu imediatamente:
	para ela.			“Aqui fala tubarão”.

**Table 2:** Stories translated and adapted by Camargo et al.^11^ for use in Brazil (original) according to plot structure, alternative stories with female (a) and male (b) characters and proposed parameters of comparability between versions.

**a. Female characters (F)**				
**Plot structure**	**Original story (f)**	** Alternative story (F1)**	**Alternative story (F2)**	**Alternative story (F3)**
Character introduction	Ana	Maria	Suzana	Regina
	Soares	da Conceição,	Borges	Cardoso,
	do Sul	do sertão	da periferia	do interior
	do Paraná,	do Ceará,	de Salvador,	de Minas Gerais,
	empregada	trabalhava	estudante	instrutora
	como faxineira	de lavadeira	de direito	de tênis
	em um prédio	para o prefeito	na Universidade	no clube
	de escritórios,	da cidade.	Federal,	da prefeitura,
Problem	relatou,	Numa manhã,	Reclamou	estava brava
	na delegacia	encontrou,	no escritório	com os funcionários
	de polícia	no bolso	da diretoria	da limpeza.
	que tinha sido assaltada	de uma calça suja,	que tinham sumido,	No dia anterior
	na Rua do Estado	uma caixinha bonita	de cima de sua mesa,	eles ficaram brincando
	na noite anterior	contendo um anel	na tarde anterior,	com as raquetes dela e
	e roubada	de ouro	os livros	depois não guardaram de volta
	em 150 Reais.	e uma nota fiscal	que ela tinha comprado.	no lugar certo
		no valor de 2.000 Reais.		
Aggravation	Ela disse que tinha 4	A joia	Ela disse que ia ter 3	Ela chamou duas
	filhinhos,	escapou de suas mãos	provas,	de suas colegas
	o aluguel não tinha sido pago	e caiu no ralo.	que ainda não tinha estudado,	para dar “uma lição” neles
	e eles não comiam		e que os exames aconteceriam	no domingo.
	há dois dias.		dali a 2 dias.	
Resolution	Os policiais	Ela pegou um galho	A diretora	Elas esconderam
	tocados pela historia da mulher	de árvore	preocupada c/ a situação da menina	os uniformes deles
	fizeram uma coleta	para tentar fisgar o anel.	emprestou livros	e só devolveram
	para ela.	Depois de 30 minutos	para ela.	quando pediram desculpas
		finalmente conseguiu recuperá-lo.		para ela.
**Comparability criteria**				
No. of items	25	25	25	25
Flesch reading ease	55.7	71.2	52.6	62.9
No. of words	66	69	67	66
No. of characters	323	318	335	334
No. of verbs	7	7	7	7
No. of nouns	15	19	17	17
**b. Male characters (M)**				
**Plot structure**	**Original story (m)**	**Alternative story (m1)**	**Alternative story (m2)**	**Alternative story (m3)**
Character introduction	Roberto	Luis	José	Alberto
	Mota	Marques	Oliveira	Lemos
	estava dirigindo	adorava	jogava	do interior
	um caminhão	escutar música	futebol	de Goiás
	Mercedes	clássica.	de salão	plantava
	numa rodovia	Seu primeiro	todo domingo	verduras e
	à noite	filho	na quadra	legumes
	no Vale	nasceu na maternidade	da cooperativa	no seu sítio.
	do Paraíba	Santa Luzia,	agrícola.	Toda semana,
	levando ovos		Estava treinando com o time	ele ia vender os produtos
	para Taubaté		para um campeonato	em Itapira
Problem	quando o eixo	e chorava muito.	quando sua chuteira	O verão
	quebrou.	Ele percebeu	desamarrou.	estava escaldante,
	O caminhão derrapou	que o silêncio acabara	O jogador tropeçou	o ar estava seco,
	caindo numa valeta	e ele não poderia mais ouvir	caindo de costas	e não havia previsão de chuva
	fora da estrada.	seus CDs.	fora do campo.	para aquela região.
	Ele foi jogado	Quando a criança		
	contra o painel	completou 8 meses		
	e se assustou muito.			
Aggravation	Não havia trânsito	ele estava desesperado,	Ele tentou levantar,	Então ele teve medo
	e ele duvidou que pudesse ser socorrido.	pois o aparelho de som	mas sua perna	que a colheita
		somente tocava musiquinhas infantis.	doía muito.	fosse prejudicada.
Resolution	Naquele instante, seu radio amador	Então, ele trocou o CD	Foi carregado de maca	Como o tempo não mudaria,
	tocou.	e colocou uma sinfonia	e levado para o vestiário.	ele resolveu tomar uma providência:
	Ele respondeu imediatamente:	de Beethoven.	O médico	Contratou
	“Aqui fala Tubarão”.	Para sua surpresa	o examinou	caminhões-pipa
		a criança se acalmou.	e viu que não havia fratura.	para regar a plantação
		E a casa virou o Paraíso	Ele ficou feliz como uma Criança	que ficou verde como Esmeralda
**Comparability criteria**				
No. of items	25	25	25	25
Flesch reading ease	60,7	65,6	66,1	58,1
No. of words	64	73	67	69
No. of characters	384	364	330	353
No. of verbs	11	11	11	9
No. of nouns	18	19	20	20

## DISCUSSION

The logical memory test is one of the most used tasks for assessing episodic memory in both clinical and research settings.^2^ In many cases, reassessment of patients is needed.^6-9^ The repeated use of this kind of test leads to learning effects due to many factors,^10^ including recall of the story content when the same story is told more than once. The use of different stories that have similar characteristics is one of the methods used to improve the validity of repeated assessments.^10^ The present study presents three alternative stories for each of the freely translated stories from the WMS-R, as these are frequently used in Brazil despite the lack of an official translation and of validation in the Brazilian population. The creation of the alternative stories was based on criteria proposed by authors who developed English versions of the task (narrative structure, emotional content, readability, number of words, etc.).^2,10,11^ The comparability of stories in terms of the plot, structure and the linguistic features were presented here, and detailed information on their psychometric equivalence can be found in the study of Martins et al.,^18^ which shows the performance of a non-clinical sample on the immediate and delayed recall of the alternative stories.

Besides the alternative stories that are similar to the Portuguese version of the WMS-R, the material presented here also includes the additional scoring criteria (gist or general themes) introduced in later versions of the WMS.^3,4^ This additional score has important clinical and experimental value, since it helps differentiate levels of impairment and allows a more detailed investigation of memory not only as an all-or-nothing phenomenon, but rather as the storage of stimuli at different levels of specificity.^20,21^

Finally, it is important to acknowledge several limitations of the present study. First of all, there are no available validated versions of the WMS stories in Portuguese for use in Brazil. Hence, the stimuli used as "models" for this study were those proposed by Camargo et al.^14^ which have been extensively used by neuropsychologists in Brazil despite not having been published in a scientific journal. The second point refers to the circumscribed aim of the present article, describing only the development of the stories, without testing their validity for clinical populations. This was necessary to show that the alternative versions were created in a similar fashion to those available in English and published in the international literature. Their equivalence to the model stories, although not presented here, was reported elsewhere.^18^ A third issue that should be pointed out is the absence of Brazilian norms for the test. For research purposes, it is recommended that matching control groups be used. However, until appropriate validation studies and norms for the WMS logical memory test are published in Brazil, the alternative stories and detailed correction criteria presented here may be used. These items may also represent a first step toward the standardization and production of norms of this task for the Brazilian population. Lastly, it must be born in mind that even with the use of alternative versions, practice effects can occur as a result of decreases in test anxiety due to familiarity with the test setting, testing procedure, and/or experimenter or clinician, as well as due to procedural learning and development of test strategies.^10^ The impact of these factors warrant attention in future studies employing the use of more than one story in the same individual, and may differ depending on the cognitive status of the patient.

## APPENDIX

**Table:** Alternative stories and complete scoring criteria for items and general themes (gist). The tables below may be used as scoring sheets for each story.

**F1. MARIA DA CONCEIÇÃO**				
**História**	**Pontuação (0 ou 1)** **Sinônimos ou ideias completas**	**Pontuação (0 ou 1)** **Temas ou ideias gerais (gist)**	**Sinônimos** **(1 ponto)**	**Não pontuar**
Maria			Maria	Outro nome
da Conceição			Conceição	Outro sobrenome
do sertão			Sertão, Interior	Outro local
do Ceará			Ceará, Cearense	Outro Estado
TEMA GERAL (GIST)			INDICAÇÃO DE QUE O PERSONAGEM PRINCIPAL ERA MULHER	
trabalhava			Trabalhava, Estava empregada	
de lavadeira			Lavadeira, Lavava roupa	Faxineira, Costureira, Doméstica, Empregada
para o prefeito			Prefeito, Casa do Prefeito	Chefe, Coronel, Deputado, Patrão, Presidente
da cidade.			Cidade	Outro lugar
TEMA GERAL (GIST)			INDICAÇÃO DE QUE O PERSONAGEM TINHA UM TRABALHO	
Numa manhã			Numa manhã, De manhã	Certa vez, Certo dia, Um dia
encontrou			Encontrou, Achou	Comprou, Ganhou
no bolso			Bolso	Qualquer outro lugar
de uma calça suja			Calça suja, Roupa suja	Calça velha, Casaco, Paletó
uma caixinha bonita			Caixinha, Caixa	Coisa, Carteira
contendo um anel			Anel, Joia, Brilhante, Aliança, (*)	Cordão, Relógio
TEMA GERAL (GIST)			INDICAÇÃO DE QUE O PERSONAGEM ACHOU UM PERTENCE ALHEIO	
de ouro			Ouro, Brilhante, Dourado (*)	Valioso
e uma nota fiscal			Nota fiscal, Recibo	Cheque, Valor em dinheiro, Folha
no valor de 2.000 Reais.			2.000 Reais	12, 100 reais, Dinheiro, 5 mil cruzados, 1 real
TEMA GERAL (GIST)			INDICAÇÃO DE QUE O OBJETO ERA CARO OU VALIOSO	
A joia			Joia, o Brilhante, Anel (*)	A caixa, Caixinha
escapou de suas mãos			Escapou, Caiu, Deixou Cair, Derrubou	Perdeu
e caiu no ralo.			Ralo	Buraco, No rio, Lago, No chão, Bueiro, Cano
TEMA GERAL (GIST)			INDICAÇÃO DE QUE O OBJETO SE PERDEU, OU QUE CAIU	
Ela pegou um galho			Galho, Pedaço de pau, Graveto, Vara, Vareta, Varinha	Pegou um palito, Garfo, Linha e anzol
de árvore			De árvore	
para tentar fisgar o anel.			Tentar fisgar, Pescar, Recuperar, Pegar	Tentou retirá-lo
Depois de 30 minutos			30 minutos	Algum tempo, 2 minutos, 2 horas, Depois de muito esforço
TEMA GERAL (GIST)			INDICAÇÃO DE QUE HOUVE ESFORÇO TENTANDO RECUPERAR O OBJETO	
finalmente conseguiu recuperá-lo.			Recuperar, Retirar, Tirar, Conseguiu, Pegar, Expressão da ideia de ter êxito.	Achou novamente
TEMA GERAL (GIST)			INDICAÇÃO DE QUE O OBJETO FOI RECUPERADO	
Pontuação Total	Max = 25	Max = 7	(*) Em caso de repetição destas palavras, pontuar apenas UMA vez cada uma.	
**F2. SUZANA BORGES**				
**História **	**Sinônimos ou ideias completas**	**Temas ou ideias gerais**	**Sinônimos** **(1 ponto)**	**Não pontuar**
Suzana			Suzana, Suzane	Outro nome
Borges			Borges	Outro sobrenome
da periferia			Periferia, Subúrbio	Morava
de Salvador			Salvador, Bahia	Outro local
TEMA GERAL (GIST)			INDICAÇÃO DE QUE O PERSONAGEM PRINCIPAL ERA MULHER	
estudante			Estudante, Cursava, Estudava, Aluna	
de direito			Direito, Advocacia	
na Universidade			Universidade, Faculdade	
Federal			Federal	
TEMA GERAL (GIST)			INDICAÇÃO DE QUE O PERSONAGEM PRINCIPAL ESTUDAVA	
reclamou			Reclamou, Denunciou, Expressão da ideia de relato queixoso.	Disse, Avisou, Declarou, Relatou, Informou, Fez boletim de ocorrência
no escritório			Escritório, Sala	Na biblioteca
da diretoria			Diretoria, Direção, Diretora (*)	Delegacia
que tinham sumido			Sumiram, Roubados, Roubaram, Roubo, Foi roubada, Furto, Expressão da ideia de sumir sem ser perdido.	Havia perdido, Perdeu
de cima de sua mesa			Mesa, Escrivaninha, Carteira	Dentro da sala de aula
na tarde anterior			Ultima tarde	Manhã, 2ª feira, Aula anterior
os livros			Livros	
que ela tinha comprado			Comprado	
TEMA GERAL (GIST)			INDICAÇÃO DE QUE O PERSONAGEM DEU FALTA DE ALGUM PERTENCE	
Ela disse que ia ter 3			Três	
provas			Provas, Testes, Exame, Avaliação (*)	
TEMA GERAL (GIST)			INDICAÇÃO DE QUE HAVERIA ALGUMA AVALIAÇÃO ACADÊMICA	
que ainda não tinha estudado			Não tinha estudado, Precisava estudar	Não havia recebido
e que os exames aconteceriam			Exames (*)	
dali a 2 dias			Dois	
TEMA GERAL (GIST)			INDICAÇÃO DE QUE O PERSONAGEM NÃO ESTAVA PREPARADO PARA A AVALIAÇÃO	
A diretora			Diretora (*)	Universidade, Professora, Chefe
preocupada com a situação da menina			Preocupada, Comovida, Sensibilizada, Com dó	Resolveu
TEMA GERAL (GIST)			INDICAÇÃO DE QUE A DIRETORA FICOU SENSIBILIZADA PELA SITUAÇÃO DA ESTUDANTE	
emprestou os livros			Emprestou, Forneceu os livros, Emprestou os livros, Deu os livros, Conseguiu os livros, Expressão da ideia de providenciar os livros.	Pediu livros emprestados, Pegou livros para estudar
para ela.			Para ela, Para a menina, Para a estudante	
TEMA GERAL (GIST)			INDICAÇÃO DE QUE AS NECESSIDADES DA ESTUDANTE FORAM ATENDIDAS	
Pontuação Total	Max = 25	Max = 7	(*) Em caso de repetição destas palavras, pontuar apenas UMA vez cada uma.	
**F3. REGINA CARDOSO**				
**História **	**Sinônimos ou ideias completas**	**Temas ou ideias gerais**	**Sinônimos** **(1 ponto)**	**Não pontuar**
Regina			Regina	Outro nome
Cardoso			Cardoso	Outro sobrenome
do interior			Interior	
de Minas gerais			Minas Gerais, Minas	Outro local
TEMA GERAL (GIST)			INDICAÇÃO DE QUE O PERSONAGEM PRINCIPAL ERA MULHER	
instrutora			Instrutora, Professora, Dá aula, Ensina	Funcionaria, Jogadora, Jogava, Coordenadora, Responsável, Trabalhava
de tênis			De tênis	
no clube			Clube	Escola, Empresa
da prefeitura			Da prefeitura, Do governo	Cidade, Do estado
TEMA GERAL (GIST)			INDICAÇÃO DE QUE O PERSONAGEM ERA ESPORTISTA OU DAVA AULA DE ESPORTE	
estava brava			Brava, Irritada, Nervosa, Bronqueada, Expressão da ideia de irritação ou raiva.	Brigaram, Brigou, Discutiu, Insatisfeita, Chateada
com os funcionários			Funcionários, Empregados	Aluna(o), Pessoas, Colegas, Crianças, Meninos, Grupo, Caboclos, Mulheres, Rapaz
da limpeza.			Limpeza, Faxina	
TEMA GERAL (GIST)			INDICAÇÃO DE QUE FICOU IRRITADA/BRAVA COM COLEGAS DO TRABALHO	
No dia anterior			Dia anterior, Expressão da ideia de véspera.	Dia seguinte
eles ficaram brincando			Brincando, Usaram, Jogando	Mexeram, Quebraram, Roubaram, Pegaram
com as raquetes dela e			Raquetes	Bola
depois não guardaram de volta			Não guardaram, Deixaram bagunçadas, Deixaram jogadas, Expressão da ideia de não devolver no lugar.	Esconderam, Perderam
no lugar certo.			Lugar certo, Correto	No armário
TEMA GERAL (GIST)			INDICAÇÃO DE QUE OUTROS PEGARAM ALGO DA PERSONAGEM PRINCIPAL, INDEVIDAMENTE	
Ela chamou duas			Duas, 2	Pediu ajuda
de suas colegas			Colega, Amiga, Outra instrutora	Mulheres
				
para dar uma “lição” neles.			Dar uma lição, Pregar uma peça, Puni-los, Dar o troco, Se vingar, Expressão da ideia de descontar.	Ajuda-la, Chamou atenção, Conversar, Dar um trato
TEMA GERAL (GIST)			INDICAÇÃO DE QUE A PERSONAGEM PEDIU AJUDA A ALGUEM PARA SE VINGAR	
No domingo			Domingo	No dia seguinte, Sábado, No outro dia, Fim de semana
elas esconderam			Esconderam	Roubaram, Lavar, Pegaram
os uniformes deles			Uniformes	Blusa, Camisas, Aventais, Raquetes, Algo que pertencia, Roupa
TEMA GERAL (GIST)			INDICAÇÃO DE QUE O PERSONAGEM FEZ ALGO CONTRA OS QUE A PREJUDICARAM	
e só devolveram			Devolveram, Entregaram, Expressão da ideia de dar de volta.	
quando pediram desculpas			Desculpas, Expressão da ideia de Perdão.	
para ela.			Para ela	
TEMA GERAL (GIST)			INDICAÇÃO DE QUE HOUVE UM PEDIDO DE DESCULPAS	
Pontuação Total	Max = 25	Max = 7		
**M1. LUIS MARQUES**				
**História **	**Sinônimos ou ideias completas**	**Temas ou ideias gerais**	**Sinônimos** **(1 ponto)**	**Não pontuar**
Luis			Luis	Outro nome
Marques			Marques, Marcos	Outro sobrenome
TEMA GERAL (GIST)			INDICAÇÃO DE QUE O PERSONAGEM PRINCIPAL ERA HOMEM	
adorava			Adorava, Gostava muito	Só gostava
escutar musica			Musica	
clássica.			Clássica, Erudita	
TEMA GERAL (GIST)			INDICAÇÃO DE QUE O PERSONAGEM PRINCIPAL GOSTAVA DE MÚSICA	
Seu primeiro			Primeiro, Primogênito, Mais velho	
filho			Filho, Bebê, Filha, Criança, (*)	
nasceu na maternidade			Nasceu na Maternidade, No Hospital, Nascimento	Casa
Santa Luzia,			Santa Luzia	Nossa senhora, Outro nome de santa
e chorava muito.			Chorava Muito, Não parava de chorar, Chorão	
TEMA GERAL (GIST)			INDICAÇÃO DE QUE O PERSONAGEM PRINCIPAL TINHA UMA CRIANÇA	
Ele percebeu			Percebeu, Se deu conta	
que o silêncio acabara			Silêncio acabara, Casa barulhenta, Fim do silêncio	Acabou a paz, Acabou paraíso, Sossego acabou
e ele não poderia mais ouvir			Ouvir, Escutar	
seus CDs			CDs, Musicas, Disco	
TEMA GERAL (GIST)			INDICAÇÃO DE QUE O PERSONAGEM NÃO PODIA OUVIR MÚSICAS POR CAUSA DE ALGO SOBRE A CRIANÇA	
Quando a criança			Criança, Bebê, Filho(a) (*)	
completou 8 meses			8 meses	18 meses, 9 meses
ele estava desesperado,			Desesperado, Em pânico, Surto, Expressão da ideia de “extrema alteração de humor”.	Estava cansado, Ficou aborrecido, Indignado, Chateado, Irritado, Não estava feliz
TEMA GERAL (GIST)			INDICAÇÃO DE QUE O PERSONAGEM ESTAVA MUITO INCOMODADO	
pois o aparelho de som			Aparelho de som, Som	Caixinha de vitrola quebrou, Pianinho, Radio
somente tocava musiquinhas infantis.			Musiquinha ou música infantil, Só ouvia musica de bebê, Musica infantil, Musica de ninar	
TEMA GERAL (GIST)			INDICAÇÃO DE QUE OUVIAM MUITA MÚSICA INFANTIL	
Então ele trocou o CD			Trocou, Expressão da ideia de “colocar outro”.	Colocou música, Colocou o cd
e colocou uma sinfonia			Sinfonia	Cd, Canção, Colocou a musica, Música
de Beethoven.			Beethoven	
TEMA GERAL (GIST)			INDICAÇÃO DE QUE O PERSONAGEM TROCOU AS MÚSICAS INFANTIS	
Para sua surpresa			Surpresa, Ficou surpreso, Se espantou	Percebeu
a criança se acalmou			Se acalmou, Choro do bebê cessou, Tudo ficou calmo, Sem barulho, Paz, Tranquila, Silêncio (*)	Filho gostou, Acordou, Criança sorriu
e a casa virou o Paraíso.			Palavra Paraíso em qualquer contexto.	
TEMA GERAL (GIST)			INDICAÇÃO DE QUE O PERSONAGEM TEVE UM RESULTADO SATISFATÓRIO	
Pontuação Total	Max = 25	Max = 8	(*) Em caso de repetição destas palavras, pontuar apenas UMA vez cada uma.	
** M2. JOSÉ OLIVEIRA**				
**História **	**Sinônimos ou ideias completas**	**Temas ou ideias gerais**	**Sinônimos** **(1 ponto)**	**Não pontuar**
José			José, Zé	Outro nome
Oliveira			Oliveira	Outro sobrenome
TEMA GERAL (GIST)			INDICAÇÃO DE QUE O PERSONAGEM PRINCIPAL ERA HOMEM	
jogava			Jogava, Jogador, Joga	Treinador
futebol			Futebol, Bola	
de salão			De salão, Salão	
todo domingo			Todo domingo, Final de semana	Todo dia, Toda semana
na quadra			Quadra, Campo	Centro, Clube, Espaço, Estádio, Time
da cooperativa			Cooperativa	União, Escola, Feira, Centro, Empresa, Time
agrícola			Agrícola, De agricultura, Agrário, Agropecuário	
TEMA GERAL (GIST)			INDICAÇÃO DE QUE O PERSONAGEM PRINCIPAL JOGAVA FUTEBOL	
Estava treinando com o time			Treinando, No treino, Praticando	Certo dia, Um dia, Jogo, Participando
para um campeonato			Campeonato, Torneio	
TEMA GERAL (GIST)			INDICAÇÃO DE COMPETIÇÃO, EVENTO ESPORTIVO	
quando sua chuteira			Chuteira, Tênis, Cadarço, Calçado, Sapato	Chinelo
desamarrou.			Desamarrou, Soltou o cadarço, Foi amarrar [pois estava solto]	Amarrar, Esqueceu de amarrar
TEMA GERAL (GIST)			INDICAÇÃO DE QUE HOUVE UM PROBLEMA COM O SAPATO	
O jogador tropeçou			Tropeçou, Escorregou, Expressão da ideia de algo que o fez cair.	Levou um tombo
caindo de costas			Caindo, Caiu	Tomou carrinho
fora do campo.			Fora do campo ou do gramado, Lateral do campo, Pra fora	Meio do campo, No chão, Quadra, Estádio
TEMA GERAL (GIST)			INDICAÇÃO DE QUE O PERSONAGEM PRINCIPAL CAIU	
Ele tentou levantar			Tentou levantar, Tentou ficar de pé	Se levantou
mas sua perna			Perna	Fratura em qualquer contexto
doía muito.			Doía, Sentiu dor	Machucou
Foi carregado de maca			Carregado, Levado, Expressão da ideia de que foi levado por outros.	
e levado para o vestiário.			Vestiário	Hospital, Pronto-socorro
TEMA GERAL (GIST)			INDICAÇÃO DE QUE O PERSONAGEM FICOU MACHUCADO E TEVE QUE SER SOCORRIDO	
O médico			Médico, Doutor, Fisioterapeuta	
o examinou			Examinou, Diagnosticou, Averiguou, Analisou, Expressão da ideia de avaliar.	Curou
TEMA GERAL (GIST)			INDICAÇÃO DE QUE O PERSONAGEM FOI EXAMINDO POR PROFISSIONAL	
e viu que não havia fratura.			Não havia fratura, Não havia quebrado, O osso estava bem	Não tinha nada, Nada havia acontecido, Não diagnosticou nada, não havia ferimento
Ele ficou feliz como uma Criança			Palavra Criança em qualquer contexto.	Feliz com a notícia, Ficou feliz, Ficou muito feliz
TEMA GERAL (GIST)			INDICAÇÃO DE QUE NÃO HOUVE FERIMENTO GRAVE	
Pontuação Total	Max = 25	Max = 8		
**M3. ALBERTO LEMOS**				
**História **	**Sinônimos ou ideias completas**	**Temas ou ideias gerais**	**Sinônimos** **(1 ponto)**	**Não pontuar**
Alberto			Alberto	Outro nome
Lemos			Lemes, Leme	Outro sobrenome
TEMA GERAL (GIST)			INDICAÇÃO DE QUE O PERSONAGEM PRINCIPAL ERA HOMEM	
do interior			Interior	
de Goiás			Goiás	Outro local
plantava			Agricultor, Plantação, Produtor, Plantador, Cultivava, Colhia	Fazendeiro
verduras e			Verduras	Alface, Frutas, Limões, Frutos
legumes			Legumes	Chuchu, Feijão,Batata, Milho, Vegetais
no seu sítio.			Sitio, Fazenda, Chácara	Horta
TEMA GERAL (GIST)			INDICAÇÃO DE QUE O PERSONAGEM TRABALHAVA COM A TERRA	
Toda semana,			Toda semana	Todo fim de semana, Todo dia
ele ia vender os produtos			Vender os produtos, Venda, Comercializava	Fazia entrega, Comprava
em Itapira.			Itapira	Outra cidade
TEMA GERAL (GIST)			INDICAÇÃO DE QUE O PERSONAGEM PRINCIPAL VENDIA O QUE PRODUZIA OU PLANTAVA	
O verão			Verão, A estação	O tempo, Clima
estava escaldante,			Escaldante, Calor, Muito sol, Sol forte, Quente, Ensolarado, Quente, Muito quente, Expressão da ideia de muito calor.	Estiagem, Terra árida, Verduras secas
TEMA GERAL (GIST)			INDICAÇÃO DE QUE O TEMPO ESTAVA MUITO QUENTE	
o ar estava seco,			Ar seco, Seca, Tempo seco, Árido, Clima árido, Expressão da ideia de secura.	Ar rarefeito, Mal tempo
e não havia previsão de chuva			Sem previsão de chuva, Escassez de chuva, Falta de chuva, Chuva não vinha	Falta de água, Acabou água
para aquela região.			Região, Área, Redondeza	
TEMA GERAL (GIST)			INDICAÇÃO DE QUE ESTAVA SECO OU SEM CHUVA	
Então ele teve medo			Medo, Preocupação, Ansioso	Achou, Pensou
que a colheita			Colheita, Safra	Perder tudo, Que a plantação suasse, Não nascesse, Safra dar errado, Perder a plantação
fosse prejudicada.			Prejudicada, Afetada	Não teria êxito
TEMA GERAL (GIST)			INDICAÇÃO DE QUE O PERSONAGEM PRINCIPAL ESTAVA PREOCUPADO	
Como o tempo não mudaria,			Tempo não mudaria, Não ia virar o tempo	Tempo ruim
ele resolveu tomar uma providência.			Resolveu tomar providencia, Decidiu agir, Expressão da ideia de resolver agir.	Teve ideia
Contratou			Contratou, Alugou, Pagou	Comprar, Usa, Ajuda
caminhões-pipa			Caminhão-pipa, Caminhão de água	Alguém, Pipa
TEMA GERAL (GIST)			INDICAÇÃO DE QUE O PERSONAGEM FEZ ALGO SOBRE A FALTA DE ÁGUA	
para regar a plantação			Regar, Pulverizar, Molhar, Fornecer água, Molhar, Aguar, Expressão da ideia de regar.	Cuidar da lavoura, Cultivar, Transportar
que ficou verde como Esmeralda.			Palavra Esmeralda em qualquer contexto.	Linda e verde, Mais verde, Ficou verde, Verde e brilhante
TEMA GERAL (GIST)			INDICAÇÃO DE QUE FOI RESOLVIDO O PROBLEMA DE FALTA DE CHUVA OU ÁGUA	
Pontuação Total	Max = 25	Max = 8		
